# Targeted Pth4-expressing cell ablation impairs skeletal mineralization in zebrafish

**DOI:** 10.1371/journal.pone.0186444

**Published:** 2017-10-17

**Authors:** Paula Suarez-Bregua, Ankur Saxena, Marianne E. Bronner, Josep Rotllant

**Affiliations:** 1 Aquatic Molecular Pathobiology Group, Institute of Marine Research (IIM-CSIC), Vigo, Spain; 2 Division of Biology and Biological Engineering, California Institute of Technology, Pasadena, California, United States of America; 3 Department of Biological Sciences, University of Illinois, Chicago, Illinois, United States of America; Universite de Nantes, FRANCE

## Abstract

Skeletal development and mineralization are essential processes driven by the coordinated action of neural signals, circulating molecules and local factors. Our previous studies revealed that the novel neuropeptide Pth4, synthesized by hypothalamic cells, was involved in bone metabolism via phosphate regulation in adult zebrafish. Here, we investigate the role of *pth4* during skeletal development using single-cell resolution, two-photon laser ablation of Pth4:eGFP-expressing cells and confocal imaging *in vivo*. Using a stable transgenic Pth4:eGFP zebrafish line, we identify Pth4:eGFP-expressing cells as post-mitotic neurons. After targeted ablation of eGFP-expressing cells in the hypothalamus, the experimental larvae exhibited impaired mineralization of the craniofacial bones whereas cartilage development was normal. In addition to a decrease in *pth4* transcript levels, we noted altered expression of *phex* and *entpd5*, genes associated with phosphate homeostasis and mineralization, as well as a delay in the expression of osteoblast differentiation markers such as *sp7* and *sparc*. Taken together, these results suggest that Pth4-expressing hypothalamic neurons participate in the regulation of bone metabolism, possibly through regulating phosphate balance during zebrafish development.

## Introduction

Recent studies have provided evidence suggesting that the central and peripheral nervous systems can directly control bone homeostasis. In this context, the hypothalamus has been recognized as a potent regulator of skeletogenesis through efferent neural signals via the sympathetic (SNS) and parasympathetic nervous systems (PSNS)[1] and/or neuroendocrine factors. Secreted neural factors can be processed through the pituitary gland or directly released into the blood stream [2–5]. Due to its ability to act as a sensor, the hypothalamus integrates physiological information from the whole body and sends inputs to bone cells that regulate bone homeostasis accordingly. The coordinated activities of the bone-forming cells (osteoblasts) and bone-resorbing cells (osteoclasts) modulate a response in bone tissue [6]. Therefore, beyond the traditional view linked to endocrine, autocrine and paracrine modulators regulating skeletal development and remodeling, bone metabolism is also regulated by neural input.

Mineralization is a key step in the formation of the bone, making it an important reservoir of calcium and phosphate that contributes to mineral balance via remodeling throughout the life span of vertebrates. Mature osteoblasts are responsible for bone matrix mineralization through the formation and accumulation of hydroxyapatite (HA) crystals. Thus, besides calcium, adequate phosphate levels are crucial for normal skeletal mineralization and healthy bone [7]. At a systemic level, the parathyroid hormone (PTH) interacts with vitamin D and fibroblastic growth factor 23 (FGF23) in the bone-parathyroid-kidney axis to control the phosphate balance in mammals [8,9]. If minerals are required, PTH also causes resorption in bone directly through parathyroid hormone receptor type I (PTH1R) [10]. Another known regulator of phosphate is PHEX (phosphate regulating gene with homologies to endopeptidases on the X chromosome), which was identified as the gene mutated in X-linked hypophosphatemic rickets (XLH) syndrome [11]. Loss of PHEX function causes hypophosphatemia, mineralization defects of bone and teeth, and abnormal vitamin D metabolism [12].

In addition, phosphate homeostasis and mineralization are locally dependent on the concentration of the two phosphate inorganic forms in the bone environment. Elevated pyrophosphate levels block the ability of phosphate to mineralize along with calcium and prevent HA formation [13,14]. The interplay of different factors expressed by osteoblasts regulates the balance between phosphate and pyrophosphate, acting as enhancers or inhibitors for mineral deposition, respectively. Along those lines, PHOSPHO1 (phosphatase orphan 1) and ENTPD5 (ectonucleoside triphosphate/diphosphohydrolase) have been shown to play an important role in phosphorus homeostasis, a critical process for an optimal calcium—phosphate product for the mineralization of bone [15,16].

Recently, we identified a new PTH family member in zebrafish, Parathyroid hormone 4 (Pth4), as a neuropeptide produced by two bilateral clusters of cells in the hypothalamus. We demonstrated that this peptide is involved in bone mass accrual through phosphate homeostasis regulation in adult zebrafish, which indicates a new functional link between brain and bone [17]. To better understand Pth4 and its role during skeletal development, we have characterized Pth4:eGFP-expressing cells and performed single-cell resolution two-photon laser ablation and confocal imaging *in vivo*. Here, we demonstrate that Pth4:eGFP-expressing cells are post-mitotic neurons and that targeted Pth4:eGFP-expressing cell ablation results in abnormal bone mineralization as well as altered expression of osteoblast differentiation markers and genes associated with impaired mineralization.

## Materials and methods

### Zebrafish

Wild-type (AB) and transgenic zebrafish lines were maintained according to Institutional Animal Care and Use Committee of the California Institute of Technology (CALTECH). Experimental procedures were approved by the Animal Experimentation Ethics Committee of CALTECH (Protocol 1346). Embryos were grown as previously described [18] and staged [19]. Embryo medium was supplemented with 0.003% (w/v) 1-phenyl-2-thiourea (PTU) to prevent pigment formation. Transgenic lines used and their abbreviations are Tg(Pth4:eGFP)iim07 = Pth4:eGFP [17] and Tg(bactin2:H2A-mCherry) = Bactin2:mCherry.

### Two-photon laser ablation and confocal imaging

Embryos were mounted, imaged, and cells ablated as previously described [20]. Pth4:eGFP; Bactin2:mCherry zebrafish were visualized throughout development as described [17] and the number of eGFP-expressing cells in the lateral hypothalamus was scored.

For cell ablation experiments, Pth4:eGFP and Bactin2:mCherry adult fish were crossed and double positive embryos were selected for experiments. Pth4:eGFP+/Bactin2:mCherry+ embryos (n = 26 per experimental group) were mounted into the mold ventral side down to allow ablation of two subsets of neural bodies. Using confocal imaging, eGFP-expressing Pth4 cells were detected and specifically selected for ablation based on red fluorescence from the ubiquitous nuclear marker. Targeted laser ablation was done with a two-photon laser at 770 nm for 10 cycles at 65% power on a Zeiss LSM 710 inverted confocal microscope with an LD C-Apochromat 40x/1.1 W Corr objective and Zen 2009 software’s ‘Regions’ tool for cell selection. Each step of the ablation process (before ablation, during ablation of selected cells, and after ablation) was captured as a single plane image, and full z-stacks were captured at the beginning and immediately at the end for each experimental embryo. Imaged embryos were analyzed to determine degree of ablation and overall health of both targeted cells and surrounding untargeted cells.

Two rounds of ablations were performed per experimental embryo at different time points (1 and 2 dpf). This protocol was chosen as the most efficient of several attempted iterations for a complete ablation process at each time point and to achieve low neuronal recovery during development. Afterwards, ablated and control larvae were captured as full z-stack images at 3 and 7 dpf to monitor recovery of the Pth4-expressing neurons. Images collected were analyzed using Imaris software (Bitplane) and exported as TIFF files.

### Immunohistochemistry

Pth4:eGFP transgenic embryos at 1 and 2 dpf were fixed with 4% PFA and washed in PBST. They were then washed in PBDT (1% BSA, 1% DMSO, 0.1% Triton X-100 in PBS, pH 7.4), blocked in 10% normal goat serum/PBDT, and incubated overnight at 4°C with primary antibodies to HuC/D (1:100; Invitrogen # A-21271) and GFP (1:400; Abcam # 6673). Further PBST washes and blocking were followed by secondary antibodies (Invitrogen) overnight at 4°C. Hoechst 34580 was added to stain nuclei (1:2500). After further PBDT and PBS washes, embryos were mounted for confocal imaging as described before [20].

### Quantitative real-time PCR

After laser cell ablation, control and ablated 7dpf larvae were homogenized in Trizol for quantitative real time PCR (qRT-PCR) analysis. Total RNA was isolated using Direct-zol^™^ RNA MiniPrep (Zymo Research) and 50 ng of RNA was reverse-transcribed according to the Maxima First Strand cDNA Synthesis Kit (Thermo Scientific) protocol. Experimental and control samples (n = 6 per group) were amplified by triplicate containing 12.5 μl of Maxima^®^ SYBR Green/ROX qPCR Master Mix (2X) (Thermo Scientific), 0.5 μl 0.2 μM of each primer, 10.5 μl nuclease free water and 1 μl of cDNA template. qRT-PCR reactions were analyzed on a 7500 Fast Real-Time PCR System (Applied Biosystems) with the following cycling conditions: initial denaturation at 95°C for 10 min followed by 40 cycles at 95°C for 15 s and 60°C for 1 min. Expression of phosphate metabolism genes and osteoblast differentiation markers was assessed using the efficiency calibrated method as previously described [21]. Relative mRNA expression levels were normalized to the housekeeping actin, beta 1(*actb1)* gene. Primer sets used for each gene are listed in Table 1.

**Table 1:** Primer sequences used for quantitative RT-PCR gene expression analysis in zebrafish.

Gene	Forward primer sequence (5´-3´)	Reverse primer sequence (5´-3´)
***pth4***	GTCTGAAGCGTCTGATCTGG	CCGGTACAGGTCGCTGAG
***actb1***	CGAGCAGGAGATGGGAACC	CAACGGAAACGCTCATTGC
***fgf23***	ATGCTGCATTCATCCGTCCT	GATGTATCTCCGCGGGTTCC
***phospho1***	TGAAAACAGGAGCAGCTGTAAA	GGGGCTGGAGATCTGCTT
***phex***	CCGTCATCACGGTATCACAA	TCTGAGCCATGGGTAAATCC
***entpd5***	ATATGCCTGAAAAGGGTGGA	TACTTCTTTGACCTCATTCAGCAG
***npt2a***	CAACACAGATTTCCCGTATCC	GCGGGCAGCTTCTCTTTG
***npt2b***	CGCCATCATTGTCAACATTC	ACTTGCAGCAACAGCAACAG
***sp7***	GACATCCGGGATCCTGGATA	TACCGTACACCTTCCCGCA
***runx2b***	CTTCAATGACCTGCGCTTTGT	TCGGAGAGTCATCCAGCTTC
***sparc***	CCCTCTGCGTGCTCCTCTTA	GCATCGCACTGCTCAAAGAA

### Skeletal staining and whole-mount in situ hybridization

Briefly, ablated and control 7dpf larvae were fixed in 2% PFA in 1X PBS for 1h at room temperature and stored at 4°C. Alcian Blue/Alizarin Red staining to visualize respectively cartilage and bone were performed essentially as described (Walker & Kimmel 2007).

For whole-mount in situ hybridization, 3dpf larvae were fixed in 4% PFA in 1X PBS overnight at 4°C and stored in 100% methanol. Whole mount *in situ* hybridization was performed as previously described [22] using digoxigenin-labeled antisense riboprobes for *sp7*, *runx2b* and *dlx2b*. First, *sp7* cDNA fragment encoding 596 pb was cloned into pGEM^®^-T easy vector (Promega) using primers 5´-GACATCCGGGATCCTGGATA-3´ and 5´-TTAAATCTCCAGCAGTCCACTG-3´. Linearized vector with NcoI were transcribed using SP6 polymerase for synthesis of the riboprobe. The *runx2b* cDNA cloned into pcDNA3 vector (gift from Crosier Lab) and *dlx2b* cloned into pBSK vector (gift from Laudet lab) were used as templates to generate the antisense RNA probes.

### Statistical analysis

Student´s t-test was used when two samples were compared and data is presented as mean ± SEM, with significance inferred from *p* values of 0.05 or less. Statistical analysis and figures were performed with PASW Statistics 18.0 and SigmaPlot 12.0, respectively.

## Results

### Hypothalamic Pth4:eGFP-expressing cells are post-mitotic neurons

Our previous work using the Pth4:eGFP transgenic reporter line showed the *pth4* promoter driving eGFP expression in two bilateral clusters of cells in the ventral part of the lateral hypothalamus. These cells project axons that extend to the anterior region of the brain as well as through the midbrain, hindbrain and spinal cord in two parallel bundles projecting caudodorsally along the body [17].

**Fig 1:**
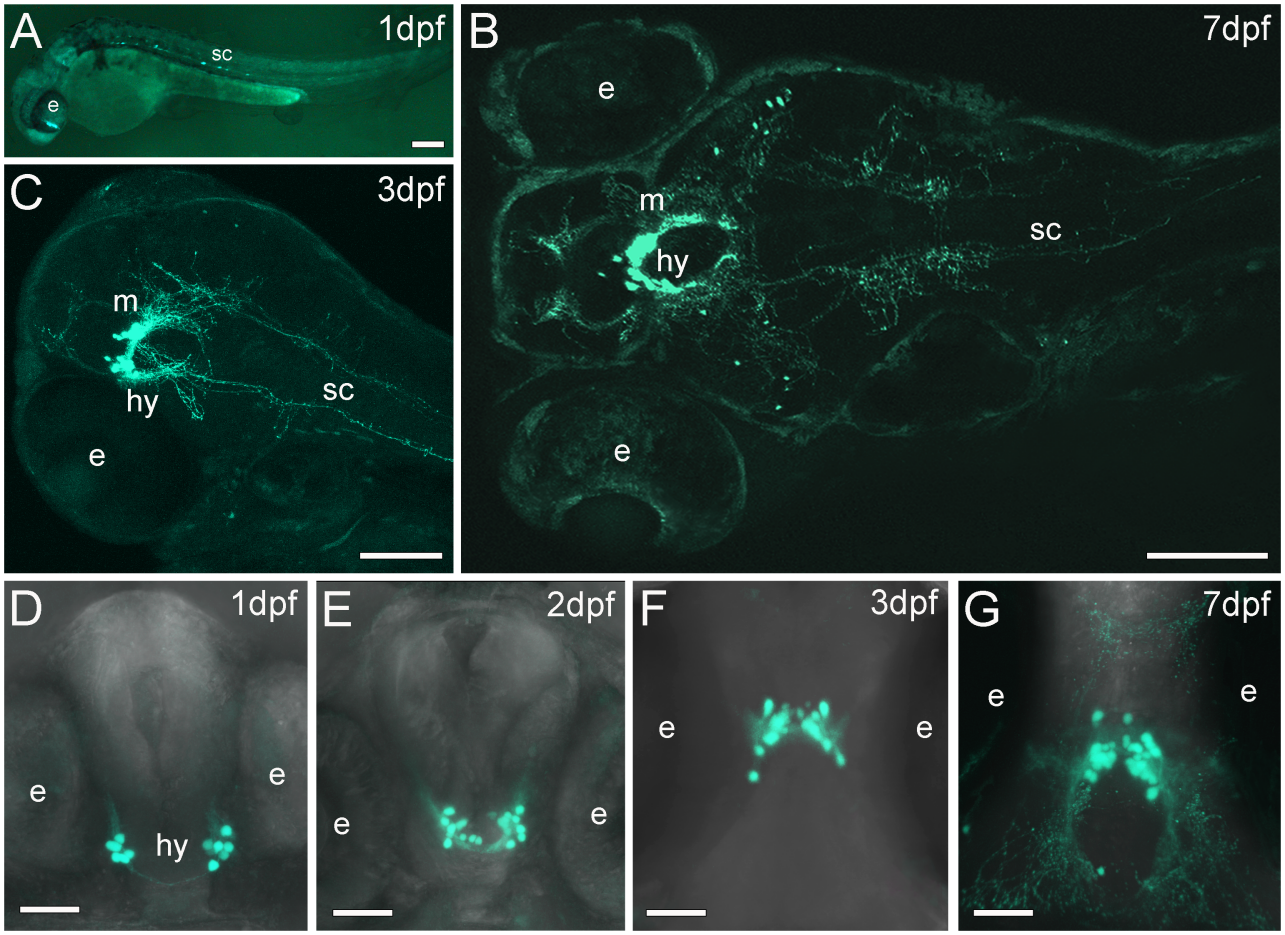
Stable transgenic Pth4:eGFP zebrafish line shows eGFP-expressing cells through development. Whole mount confocal imaging showing eGFP-expressing cells and axonal projections at 1, 3 and 7 dpf (A, B and C). Confocal z-stack projection showing two clusters of Pth4:eGFP-expressing cells on the lateral hypothalamus in a ventral view (D, E and F) or dorsal view (G). The total number of eGFP cells was measured by analyzing each 2μm thick z-plane slice from the full confocal z-stack projection in each stage of development: (D) 14±2 eGFP cells at 1dpf, (E) 22±2 eGFP cells at 2dpf, (F) 29±2 and (G) 33±1 eGFP cells at 3 and 7dpf, respectively). Note that the number of cells increases over time and axonal projections become more abundant and branched (B, G). Two groups of Pth4:eGFP-expressing cells move gradually from a caudo-ventral to a more rostro-dorsal position. Abbreviation: e, eye; hy, hypothalamus; m, midbrain; sc, spinal cord. Scale bars: 100 μm (A-C) 50 μm (D-G).

To perform a detailed analysis of the development of these neurons and the formation of connections, we used confocal imaging to observe the Pth4 expressing cells as a function of developmental time in Pth4:eGFP transgenic zebrafish. By 1 dpf, we found 13–16 eGFP-expressing cell bodies forming two separate and distinct groups in the lateral hypothalamic area and incipient neuronal projections extending to the anterior region of the brain (Fig 1A and 1D). From 2 dpf onwards, the two bilateral clusters of cells were closer together and moved to a rostral and slightly dorsal position (Fig 1E–1G). The number of cells observed was 20–25 at 2 dpf (Fig 1E), 27–30 at 3 dpf (Fig 1B and 1F), and 32–34 by 7 dpf (Fig 1C and 1G). Cell projections were visualized ventrally between the two groups of eGFP positive cells (Fig 1D–1F). Over time, the number of neuronal projections progressively increased, and at 7 dpf, we observed a complex network of axonal fibers from a dorsal view (Fig 1C and 1G).

**Fig 2:**
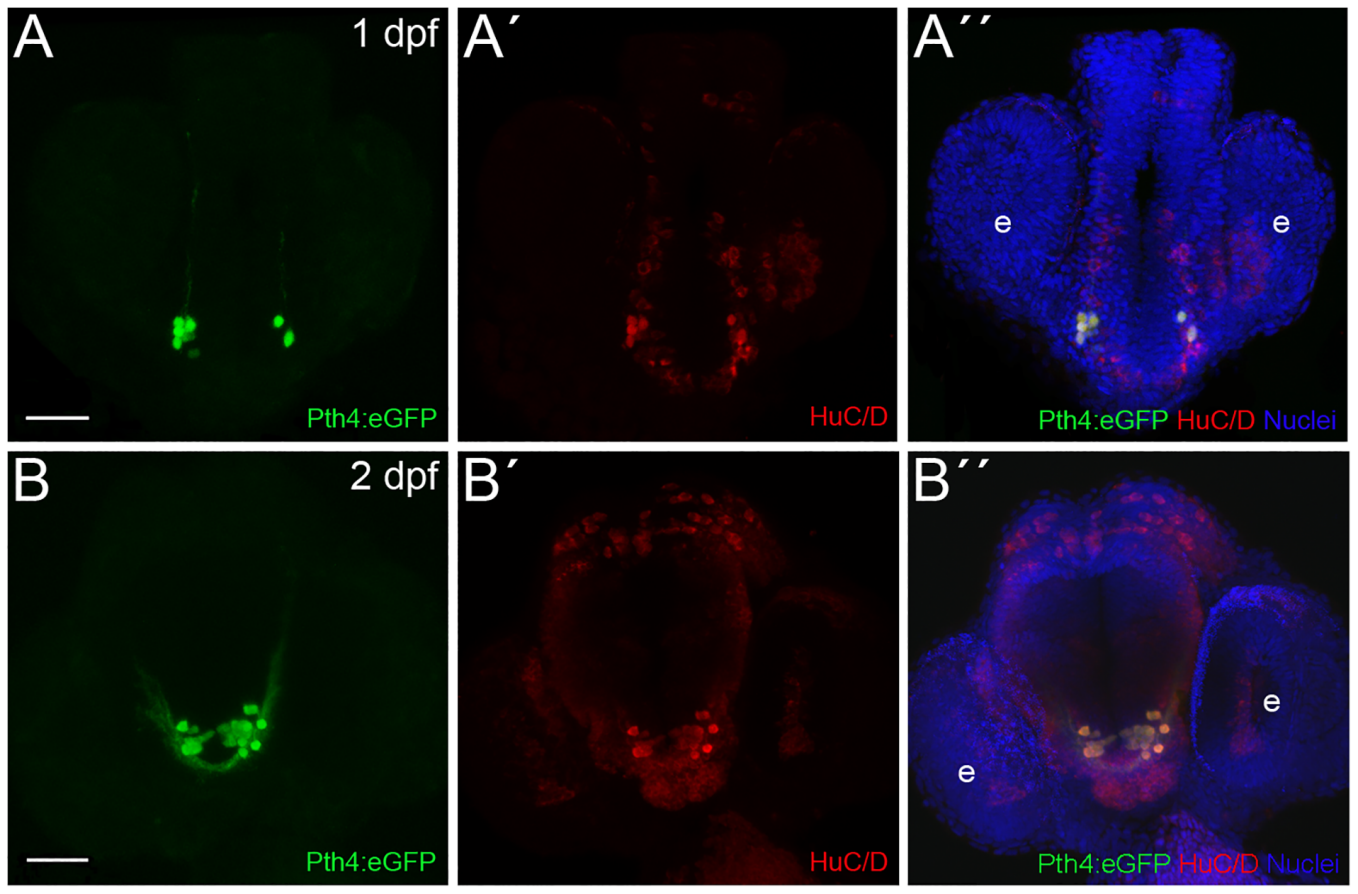
Zebrafish Pth4:eGFP-expressing cells are post-mitotic neurons. Double inmunostaining in Pth4:eGFP transgenic embryos using anti-eGFP antibody (A and B) and anti-HuC/D antibody (A´and B´) shows complete co-localization at 1 and 2 dpf (A´´ and B´´). Ventral views with anterior to the top. Pth4:eGFP: green; HuC/D: red; nuclear stain: blue. Abbreviation: e, eye. Scale bar: 50 μm.

In order to further characterize these eGFP-expressing cells, we performed immunostaining on Pth4:eGFP-positive embryos at 1 and 2 dpf using HuC/D antibody, a marker for young post-mitotic neurons [23]. The results showed that eGFP expression co-localized with that of HuC/D in every cell from the two subsets, confirming that these eGFP-expressing cells are post-mitotic neurons (Fig 2A and 2B).

### Two-photon laser ablation of Pth4:eGFP+/Bactin2:H2A-mCherry+ neurons severely reduces *pth4* gene expression in larvae

**Fig 3:**
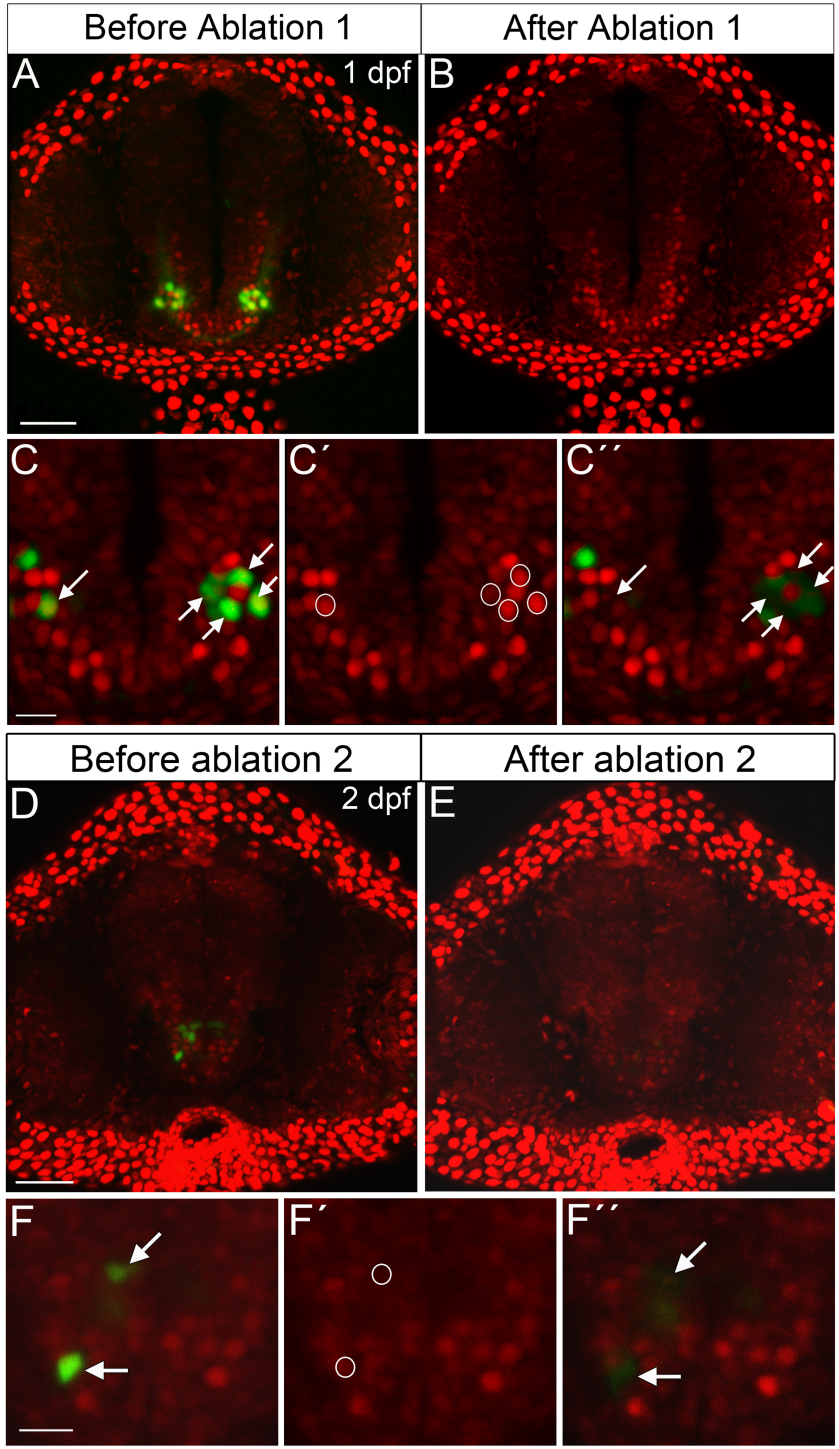
Two-photon laser ablation of Pth4:eGFP-expressing cells at 1 dpf and 2 dpf. Before ablation 1 and 2, Pth4:eGFP^+^/Bactin2:H2A-mCherry^+^ transgenic embryo shows two bilateral clusters of neurons in the hypothalamic area (A and D). After several rounds of ablation the eGFP-expressing cells are completely eliminated (B and E). Ablation processes example (C-C´´and F-F´´): (C and F) arrows indicate eGFP-expressing cells chosen for ablation; nuclear region of interest for targeted elimination of Pth4:eGFP neurons is defined by circles, based on red fluorescence from nuclear marker Bactin2 (C´ and F´); immediately after ablation Pth4:eGFP neurons are eliminated (marked by arrows in C´´ and F´´). Note that the untargeted cells around the eGFP-expressing cells ablated are intact (C´´ and F´´). A confocal z-stack projection (A, B, D and E) or single 2 μm thick z-plane slice (C-C´´ and F-F´´) are shown. Scales bars: 50 μm (A, B, D and E); 15 μm (C-C´´ and F-F´´).

**Fig 4:**
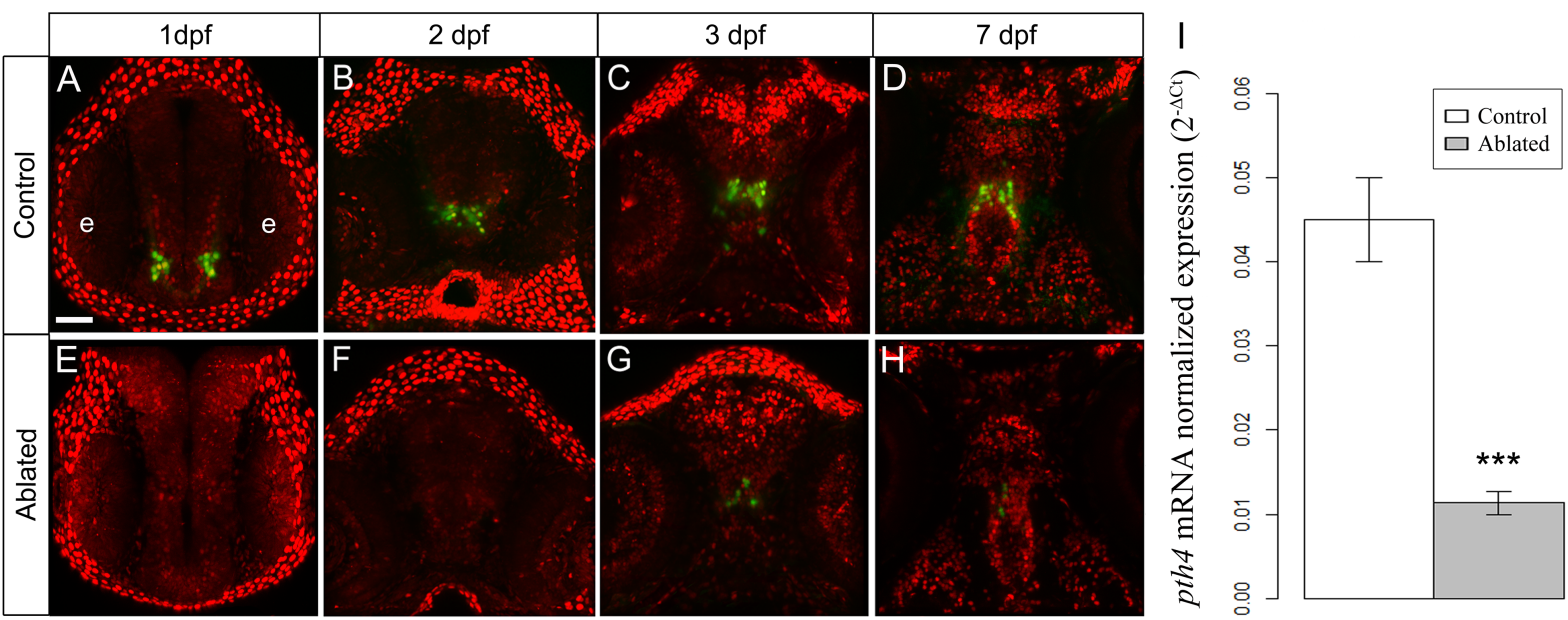
Illustration showing the development of the two clusters of Pth4:eGFP neurons in control (A-D) and ablated larvae (E-H) from 1 to 7 dpf. After first and second round of ablation, 1 and 2 (at 1 and 2 dpf, respectively) the eGFP-expressing cells are totally eliminated (E and F); at 3 dpf and 7 dpf a low number of Pth4:eGFP-expressing neurons recover (G-H), but there remains significantly decreased *pth4* gene expression (***p< 0.001) in 7 dpf ablated larvae (I). Results normalized to *actb1* are expressed as mean ± SEM. Confocal z-stack projections are shown. Abbreviation: e, eye. Scales bars: 50 μm.

We next performed two-photon laser ablation of Pth4:eGFP and Bactin2:H2A-mCherry double-positive cells during early development in order to down-regulate the *pth4* gene expression and investigate whether elimination of Pth4:eGFP-expressing neurons result in skeletal abnormalities. First, the ablation process was performed at 1 dpf, and approximately 13–16 cells were eliminated per experimental embryo. Despite complete Pth4:eGFP-positive neuronal ablation, we observed that this early and single round of ablation at 1 dpf was followed by partial replenishment of Pth4:eGFP-expressing cells at 2 dpf, which increased over time until similar levels were found to those in 7 dpf control larvae, suggesting some regenerative ability. Our observations suggested that the production of new eGFP-expressing cells decreased as the embryo developed. Therefore, we next attempted cell ablation at 2 dpf and found that the capacity of Pth4-expressing cells to recover after ablation was markedly reduced compared with that observed in 1 dpf experiments. However, nearly double of the number of Pth4-expressing neurons were present at 2 dpf versus 1dpf, making timely single cell ablation more difficult. To optimize the specific elimination of Pth4-expressing cell bodies without impairing surrounding cells, we decided to perform two rounds of laser ablation per embryo, one each at 1 and 2 dpf (Fig 3). The first round of laser ablation of Pth4:eGFP/Bactin2:H2A-mCherry double-positive cells resulted in the elimination of 13–16 cell bodies at 1 dpf embryos (Fig 3A and 3B). Targeted cells expressing eGFP and mCherry markers were detected, selected and ablated (Fig 3C–3C´´) until elimination of Pth4:eGFP-expressing cells was complete (Fig 3B). Prior to the second round of laser ablation at 2 dpf, we observed ~10 eGFP-expressing cells in ablated embryos (Fig 3D) that were selected and ablated (Fig 3E) in a second round of laser ablation (Fig 3F–3F´´). Afterwards, no eGFP-positive cells were detected in some larvae at 3dpf or as few as 1 to 7 eGFP-expressing cells at 3 dpf (Fig 4G), but never more than 10 cells by 7 dpf (Fig 4H). Therefore, this two-round ablation strategy resulted in a significant reduction of Pth4:eGFP-expressing cells to a level around three- to four-fold less than that in control embryos at 7dpf (Fig 4D and 4H). To further validate the efficiency of this method and to determine whether the laser ablation of eGFP-expressing cells caused a reduction in *pth4* gene expression, we quantitatively analyzed *pth4* expression levels by qRT-PCR analysis. The results revealed significantly (***p<0.001) reduced *pth4* gene expression in ablated larvae at 7 dpf when compared with non-ablated controls (Fig 4I).

### Bone mineralization is impaired in larvae after early Pth4:eGFP-expressing cells ablation

**Fig 5:**
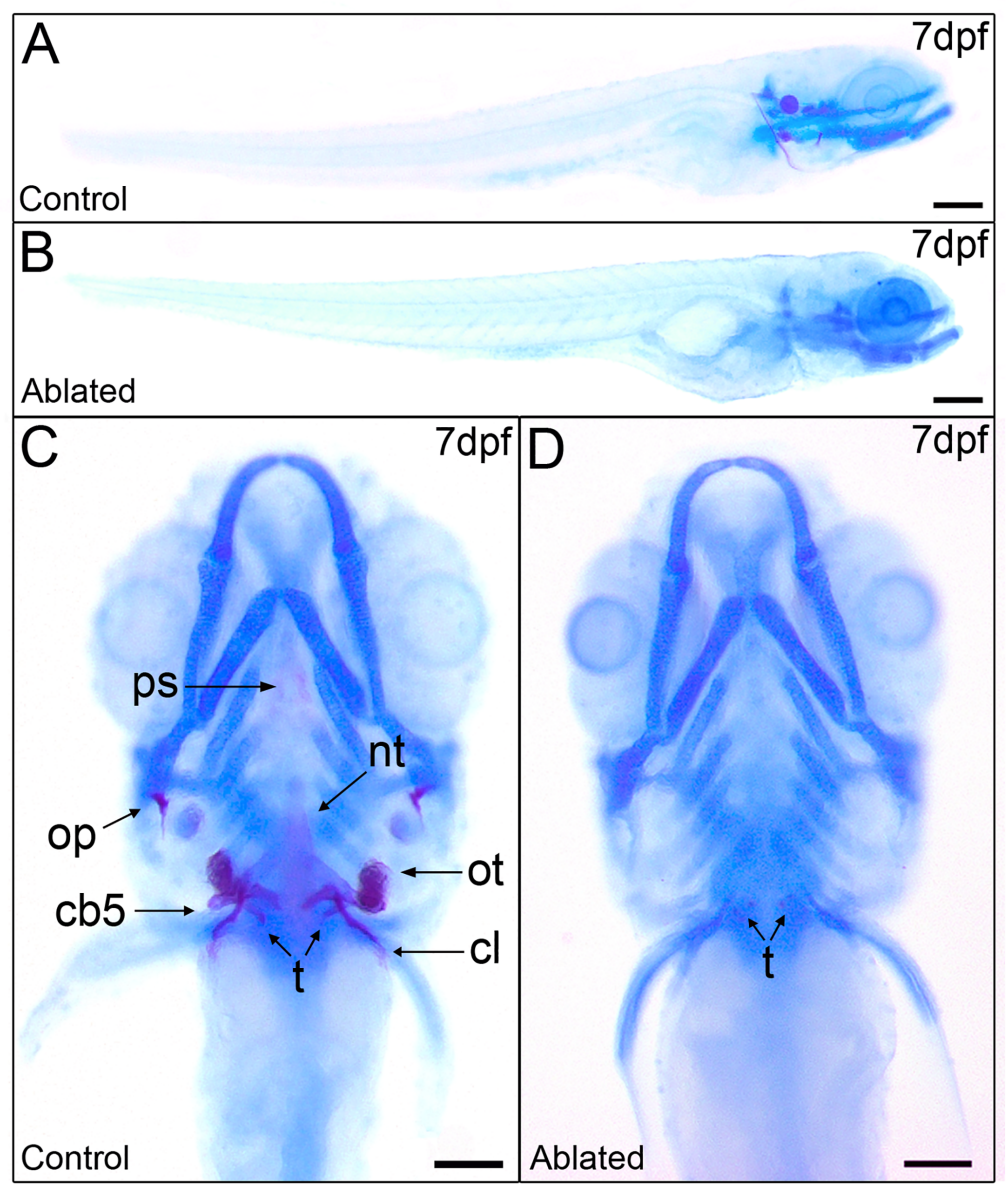
Ablated larvae exhibit impaired mineralization of the craniofacial skeleton but normal cartilage development. Skeletal staining in 7 dpf control larvae (A and C) shows calcification of the craniofacial bones unlike ablated larvae (B and D) with a widely non-mineralized skeleton except an incipient mineral deposit on teeth. Lateral views (A and B) and ventral views (C and D). Abbreviations: ps, parasphenoid; nt, notochord tip; op, operculum; ot, otolith; cb5, ceratobranchial arch 5; t, teeth; cl, cleithrum. Scale bars: 100 μm.

Since previous functional characterization of Pth4 demonstrated its involvement in bone mineral homeostasis in adult zebrafish, we asked whether Pth4:eGFP-expressing neuronal ablation would affect skeletal development and mineralization. Skeletal staining was performed using Alcian Blue/Alizarin Red to stain cartilage and mineralized bone matrix on 7 dpf ablated and control larvae (Fig 5). There was no change in cartilage morphology (Fig 5A–5D), a useful internal control for normal embryonic development. However, ablated larvae displayed a lack of some common mineralized structures such as notochord tip (nt), operculum (op), otolith (ot), cleithrum (cl) and ceratobranchial arch 5 (cb5) (Fig 5B and 5D), which would normally be calcified at this developmental stage (Fig 5A and 5C). Furthermore, we detected a slight mineral deposit on teeth (t) (Fig 5D).

### Ablated larvae show specific effects on markers of bone homeostasis

**Fig 6:**
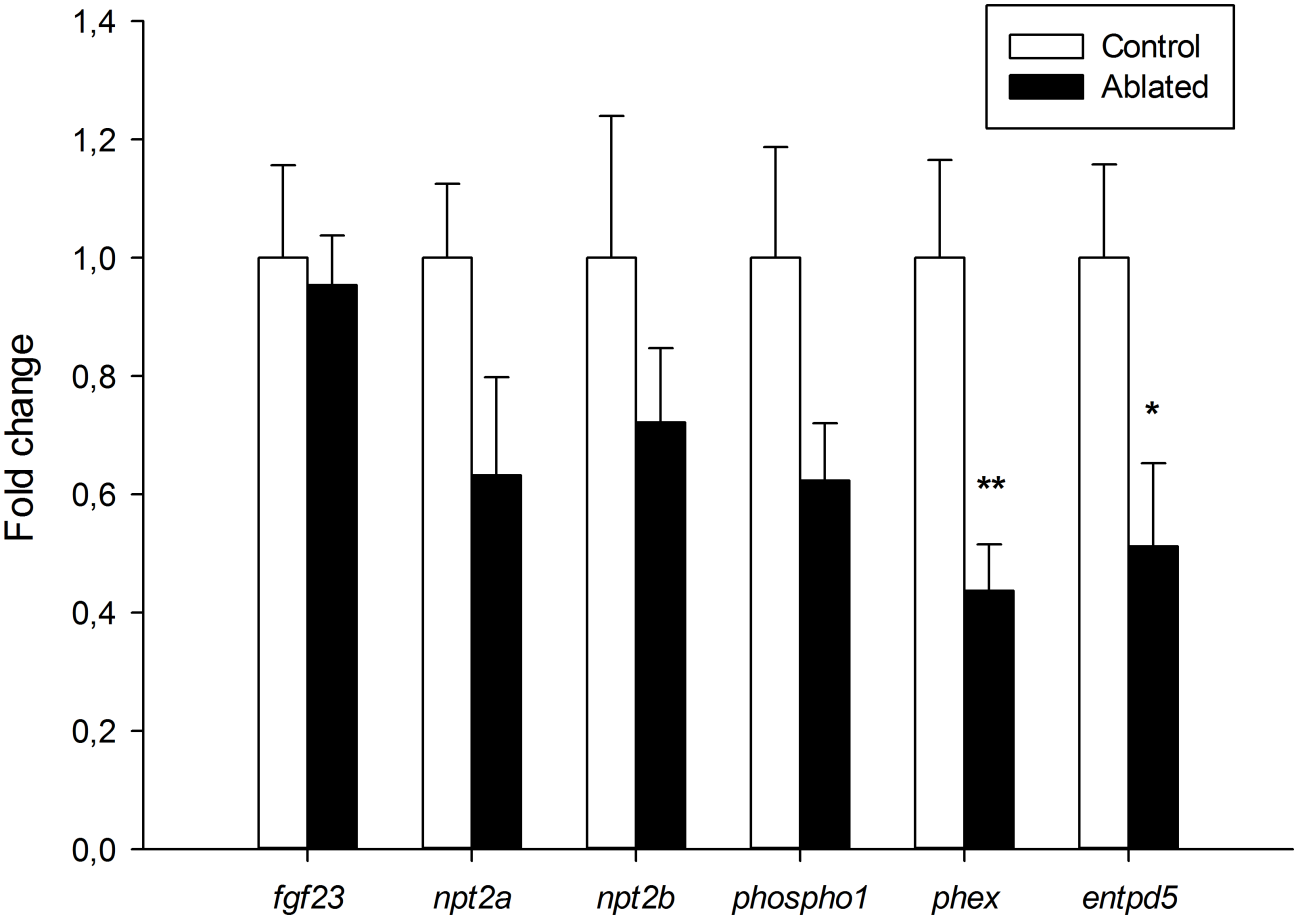
Mineralization and phosphate metabolism markers are altered post-ablation. qRT-PCR on 7 dpf larvae shows significant down-regulation of *phex* (**p<0.025) and *entpd5* (*p<0.05) expression in ablated larvae (black bars) compared with controls (white bars). Results normalized to *actb1* are expressed as mean ± SEM.

The lack of mineralization in most craniofacial structures in ablated zebrafish larvae at 7 dpf prompted us to evaluate several bone mineral homeostasis gene markers. It is known that Pth4 gain-of-function leads to a dysregulation of genes that are involved in regulating phosphate homeostasis and bone mineralization in adult fish [17]. To investigate the relationship between the alteration of mineralization in ablated larvae and gene expression of phosphate metabolism markers, we performed quantitative RT-PCR analysis in ablated and control larvae at 7 dpf. We analyzed the gene expression levels of *fgf23*, *npt2a* and *npt2b*, which are well-known regulators of systemic phosphate maintenance [9,24], and *phospho1*, *phex and entpd5* that locally facilitate skeletal mineralization by promoting phosphate availability through paracrine/autocrine pathways in bone [15,16,25]. Interestingly, we found significant down-regulation of *phex* and *enpd5* in 7 dpf ablated larvae when compared to control larvae gene expression (Fig 6). Transcript levels of *fgf23*, *npt2a*, *npt2b* and *phospho1* remained unchanged (Fig 6).

### Early Pth4:eGFP-positive neuronal ablation affects sequential gene expression during the process of osteoblast differentiation

**Fig 7:**
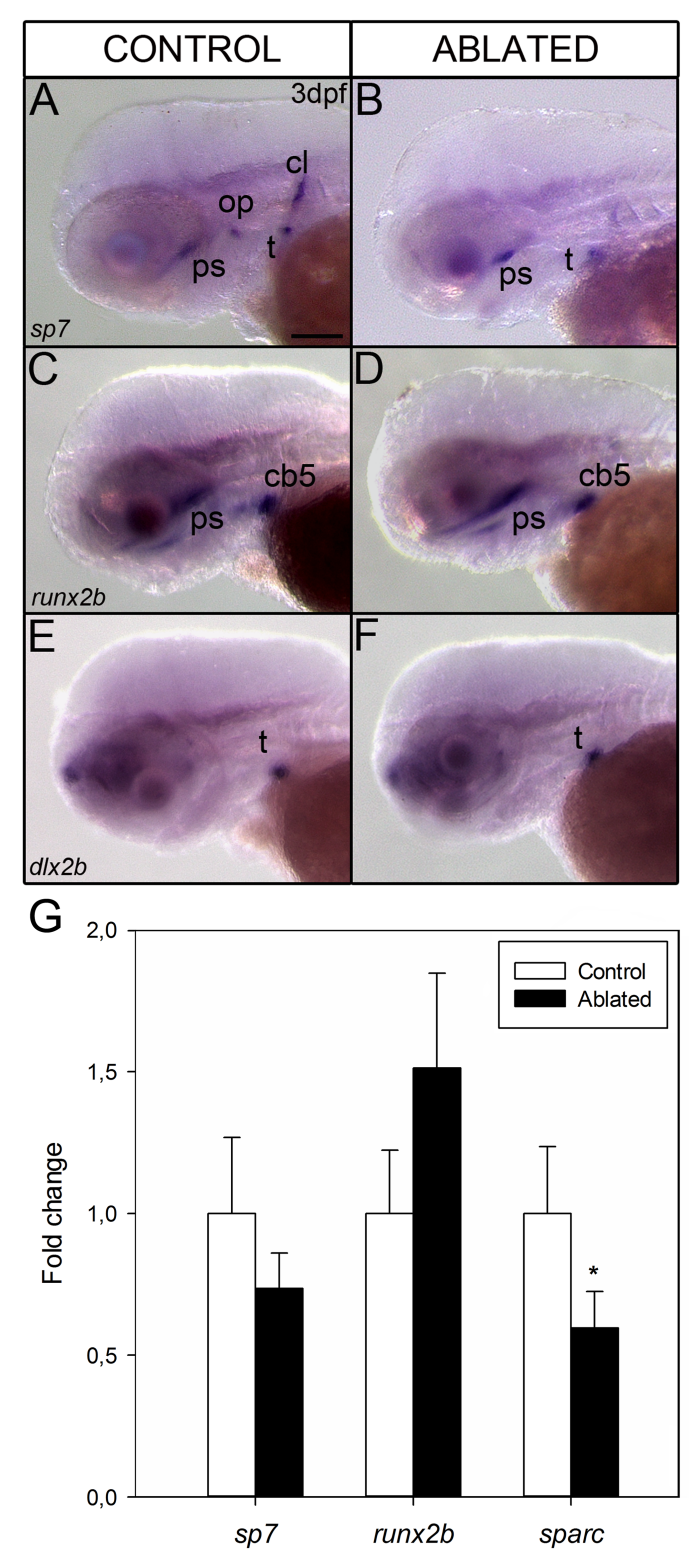
Whole mount *in situ* hybridization shows the gene expression pattern of osteoblast differentiation markers at 3 dpf in control (A, C and E) and ablated larvae (B, D and F) from lateral view. Expression of *sp7* is detected in operculum, cleithrum, teeth and parasphenoid (A). Note the absence of expression in cleithrum and operculum in ablated larvae (B). *runx2b* and *dlx2b* display the same expression pattern before ablation (C, E) and after ablation (D, F). Quantitative RT-PCR analysis of early (*runx2b*), intermediate (*sp7*) and late (*sparc*) stage markers of osteoblast differentiation in 7 dpf ablated (black bars) and control larvae (white bars) (G). Results show significant down-regulation of *sparc* gene expression (*p <0.05) when compared with controls. Results normalized to *actb1* are expressed as mean ± SEM. Abbreviations: ps, parasphenoid; op, operculum; cb5, ceratobranchial arch 5; t, teeth; cl, cleithrum. Scale bars: 100 μm.

As in mammals, zebrafish require orchestrated gene expression during all stages of osteoblastogenesis [26]. We asked whether ablation of Pth4:eGFP-expressing neurons in zebrafish would alter the differentiation of osteoblasts and perhaps prevent proper mineralization of the head bony structures. To test this hypothesis, we examined expression of osteoblast lineage markers by whole mount *in situ* hybridization in 3 dpf ablated and control larvae. Transcript expression of osterix (*sp7*) was significantly decreased in the operculum and cleithrum (Fig 7A and 7B). *Sp7* is a marker for the intermediate stage of osteoblast differentiation [26] and this reduced gene expression suggests an alteration in the maturation process of osteoblasts. No significant changes were found in runt-related transcription factor 2b *(runx2b)* gene expression, which is a marker of early osteoblast differentiation (Fig 7C and 7D) [26,27]. Since skeletal staining showed normal tooth mineralization in ablated larvae, we also tested *dlx2b*, a dental epithelium marker required for tooth formation [28]. No differences were found in distal-less homeobox 2b (*dlx2b)* gene expression among ablated and control larvae (Fig 7E and 7F).

To further investigate whether the abnormal craniofacial mineralization observed in 7 dpf ablated larvae was caused by the absence of mature osteoblasts, as suggested by the decrease of *sp7* gene expression at 3 dpf, or if instead mature osteoblasts were present but not mineralizing, we also analyzed *sp7*, *runx2b* and *sparc* transcript levels at 7 dpf by qRT-PCR (Fig 7G). We found no significant differences in *sp7* and *runx2b* gene expression levels. However, a known marker of mineralizing osteoblasts, *sparc* [26], showed a significant down-regulated expression in ablated zebrafish larvae (Fig 7G).

## Discussion

Bone formation and mineralization are dynamic processes that begin during larval stages and continue throughout adult life, so that it is expected that most of cellular and molecular mechanisms involved are common for early development and skeletal modeling and remodeling [29]. In the present study, we found that Pth4:eGFP-expressing cells are hypothalamic post-mitotic neurons, and early laser ablation of these cells caused a severe alteration in the mineralization of craniofacial bones in larvae as well as a dysregulation of the expression of some phosphate metabolism and bone mineralization gene markers.

The Pth4:eGFP transgenic line and confocal live imaging allowed us to visualize Pth4:eGFP-expressing cells during zebrafish development. Immunostaining revealed Pth4:eGFP-expressing cells as post-mitotic neurons. By observing the two clusters of neurons in the ventral hypothalamus as a function of time, we detected a progressive increase in the number of neurons from 13–16 cells at 1 dpf to 27–30 cells at 3 dpf to 32–34 cells at 7dpf. In the first week of development, bilateral clusters of neural cells changed from a caudo-ventral to a more rostro-dorsal position as projections progressively increased and branched. The increase observed in the number of these differentiated neurons does not appear to be due to cell proliferation of eGFP-expressing cells, given that these cells are post-mitotic neurons and they have possibly lost their ability to divide. Instead, embryonic neural stem cells or their progeny might be recruited from proliferative areas in the brain in order to migrate to this particular hypothalamic area where they would differentiate into post-mitotic Pth4 neurons [30].

The study of novel neural pathways that control bone mass sheds light on complex skeletal metabolism and its multi-scale regulation. A growing number of peptides and hormones synthesized in the hypothalamus have been shown to participate in the central control of the bone mass in vertebrates (reviewed in [5]). Here, we used a functional approach to evaluate whether the targeted deletion of Pth4:eGFP-expressing cells at early stages affected skeletal development and bone mineralization. The cell ablation strategy based on two rounds of ablations at 1 and 2 dpf allowed us to perform specific elimination of the vast majority of Pth4:eGFP-expressing cells without damaging neighboring cells. We detected some regenerative ability possibly resulting in production of some new Pth4:eGFP-expressing neurons, but the degree of reduction was sufficient to promote a significant down-regulation of *pth4* gene expression.

We find that early laser ablation of Pth4:eGFP-expressing neurons results in important changes in the mineralization of craniofacial structures such as the notochord tip, operculum, otolith and ceratobranchial arch 5 in 7dpf larvae. Since mineralization requires a controlled expression of phosphate regulating genes at systemic level but also in the bone environment, we analyzed some phosphate homeostasis markers at 7dpf larvae after ablation. The ablated fish larvae with impaired mineralization display a significant decrease of *phex* and *entpd5* gene expression. In mammals, *PHEX* is expressed by osteoblasts and osteocytes in bone and by odontoblasts in teeth [31]. PHEX deficiency results in defective bone mineralization and renal phosphate wasting [12]. It has been suggested that *PHEX* acts directly or indirectly upstream of *FGF23* in bone, because the loss of *PHEX* leads an increase of *FGF23* and hypophosphatemia [25,32]. Although our results showed a significant decrease in *phex* expression levels in ablated zebrafish larvae, *fgf23* gene expression did not change, which suggests that *phex* could also regulate skeletal mineralization through other mechanisms. The small ASARM peptide (acidic serine aspartate-rich MEPE-associated peptide) is a mineralization inhibitor which is hydrolyzed by *PHEX* for bone homeostasis maintenance [33]. Other crucial factor for bone formation and mineralization is *entpd5*. Zebrafish *entpd5* has been identified as a promoter for inorganic phosphate availability in the bone microenvironment [15]. Although the differential gene expression analysis showed changes in the levels of *entpd5* and *phex* transcripts, we did not detect significant alterations in the expression levels of other phosphate homeostasis markers. Thereby, we should keep in mind that the dilution effect by using mRNA from whole larvae rather than discrete tissues could underestimate the expression levels of low copy gene markers. Parathyroid hormone (PTH) family members (*i*.*e*. PTH and parathyroid hormone like hormone-PTHLH) directly regulate the expression of numerous genes in osteoblastic cells to control bone mineral metabolism and skeletal development [34,35]. Loss of function of *pthlha* and *pthlhb* in zebrafish caused deformities in craniofacial cartilage structures and delay or inhibition of bone mineralization through its downstream target *runx2* [36]. Also, Pthlh participates in mineralization of scales promoting the down-regulation of *sparc* gene expression for calcium mobilization [37]. Because our results indicate a strong alteration in the craniofacial skeleton mineralization, we examined whether *pth4* transcript down-regulation, due to laser ablation of Pth4:eGFP-expressing cells, could affect sequential gene expression during osteoblast differentiation. In this context, the bony structures-forming cells in ablated larvae could be mature osteoblasts unable to mineralize the bone matrix or, on the contrary, immature osteoblasts. The osteoblast differentiation process from osteoprogenitor cells to mature osteoblasts is characterized by an orchestrated gene expression, which seems to be conserved between mammals and fish [26]. Our gene expression analysis of osteoblast lineage markers using both whole mount *in situ* hybridization and quantitative RT-PCR revealed no differences in *runx2b* expression, while *sp7* gene expression, decreased in 3 dpf ablated larvae, recovered to normal levels by 7 dpf. Although *RUNX2* seems to act upstream of *SP7* during bone formation [38], other studies showed that *SP7* regulates osteoblast differentiation in a *RUNX2*-independent pathway [39]. On the other hand, *runx2b* has been shown as a key modulator for chondrocyte differentiation and proliferation in zebrafish [40], which may explain the normal development of craniofacial cartilage in ablated larvae. Additionally, by 7 dpf a significant decrease in *sparc* transcript levels was found, suggesting a possible alteration in the ability of mature osteoblasts to mineralize. Taken together, these results reveal a delay in the sequential expression of osteoblast differentiation markers after laser ablation of Pth4:eGFP-expressing cells. In the short term, the osteoblast maturation process was disturbed, resulting in osteoblasts unable to mineralize the bone matrix in 7 dpf ablated zebrafish larvae. Therefore, cartilage and bone development do not seem to be affected but mineralization is uncoupled from bone formation.

In conclusion, we show that hypothalamic Pth4:eGFP-expressing cells are differentiated neurons, and the decrease of *pth4* transcript levels caused by laser ablation of Pth4:eGFP-expressing neurons causes a significant reduction of *phex* and *entpd5* gene expression as well as delayed expression of genes involved in osteoblast differentiation such as *sp7* and *sparc*. Additionally, ablated zebrafish larvae exhibit impaired craniofacial bone mineralization likely due to the presence of non-mineralizing osteoblasts. Therefore, our results provide new evidence regarding neuronal regulation of the bone mineralization during fish skeletogenesis.
